# Cognitive impairment in psoriasis patients: a systematic review of case–control studies

**DOI:** 10.1007/s00415-022-11317-2

**Published:** 2022-08-09

**Authors:** Daniel Pankowski, K. Wytrychiewicz-Pankowska, W. Owczarek

**Affiliations:** 1 University of Warsaw, Stawki 5/7, 00-183 Warsaw, Poland; 2 University of Economics and Human Sciences in Warsaw, Warsaw, Poland; 3 Department of Dermatology, Military Institute of Medicine, Warsaw, Poland

**Keywords:** Cognitive impairment, Psoriasis, Systematic review

## Abstract

**Introduction:**

Cognitive impairment in chronic diseases such as psoriasis is an increasing clinical challenge.

**Objective:**

To assess the frequency and extent of difficulties in cognitive functioning in people with psoriasis compared to healthy people.

**Patients and methods:**

The systematic review was carried out on the 23rd July, 2021 by two trained psychologists resulting in a selection of 11 studies on 971 patients with psoriasis and 10,242 controls.

**Results:**

A review of the studies showed irregularities in many cognitive domains, including working memory processes, executive functions, long-term verbal memory, attention, and the visuospatial domain. Depending on the methods used to assess cognitive dysfunctions and the characteristics of patients in different studies, large differences in the frequency of cognitive impairment in patients with psoriasis were observed, ranging from 0 to 91.9%.

**Conclusions:**

The authors conclude that there is a need for longitudinal studies to identify factors important for the development and persistence of cognitive impairment in psoriatic patients.

**Supplementary Information:**

The online version contains supplementary material available at 10.1007/s00415-022-11317-2.

## Introduction

Psoriasis is a chronic inflammatory disease whose etiopathogenesis has not been fully elucidated [1]. It affects women and men to a similar extent [2], and its incidence depends on region [3]. This disease is associated with an increased risk of comorbidities such as cardiometabolic diseases [4]. Psoriasis also significantly affects the functioning of patients, reducing their quality of life [5] and numerous publications indicate a high frequency of depressive symptoms, alcohol misuse, and anxiety among those suffering from it [6].

However, little space has been devoted to the assessment of difficulties in cognitive functioning in psoriatic patients [7]. There is much literature on cognitive impairment (CI) in other diseases, such as cancer [8] or chronic obstructive pulmonary disease [9], indicating its negative impact on the quality of life of patients [10]. The literature indicates that such difficulties may be related to, inter alia, mood [11] and other psychological factors such as stress [12]. Earlier studies indicated a relationship between cognitive impairment and the severity of depressive symptoms, e.g., in the group of elderly people [13], as well as in clinical groups [14]. Due to the very high prevalence of depressive symptoms in the group of patients with psoriasis [15], it was decided to also control this variable in the systematic review.

Another factor that may be associated with a higher risk of CI is the use of cytostatic treatment: many researchers link this with the phenomenon of "chemo-brain"/cancer-related cognitive dysfunction [16]. However, the doses of drugs such as methotrexate (MTX) used in dermatology are much lower than those used in, for example, oncology or rheumatology.

Therefore, we performed a systematic review and meta-analysis of data from previously published studies to determine the severity of cognitive impairment in people diagnosed with psoriasis compared to healthy people and to estimate the frequency of this phenomenon. Additionally, we attempted to identify factors that may be moderators of the severity and frequency of cognitive impairment, in particular focusing on the severity of depressive symptoms.

## Methods

### Search strategy

The database search was carried out on the 23rd July, 2021 by two psychologists (DP and KWP) who have undergone specialist training in this field with the PRISMA protocol [17]. The reviewers searched EBSCO (Academic Research Source eJournals, Academic Search Ultimate, APA PsycArticles, ERIC, Health Source—Consumer Edition, Health Source: Nursing/Academic Edition, MEDLINE), Pubmed, Science Direct, the Cochrane Library, and the National Technical Information Service using the following keywords: “psoriasis AND cognitive impairment AND/OR cognitive decline AND/OR MCI AND/OR cognitive difficulties AND/OR neuropsychological”; “psoriasis AND MMSE AND/OR MoCA AND/OR attention AND/OR memory AND/OR executive functions”. Google Scholar was also searched and each record was carefully analyzed for methodological correctness. No time restrictions were used in the search and the ethnicity of participants was not taken into account. The reference lists of relevant articles were also reviewed.

### Selection criteria

**Fig. 1:**
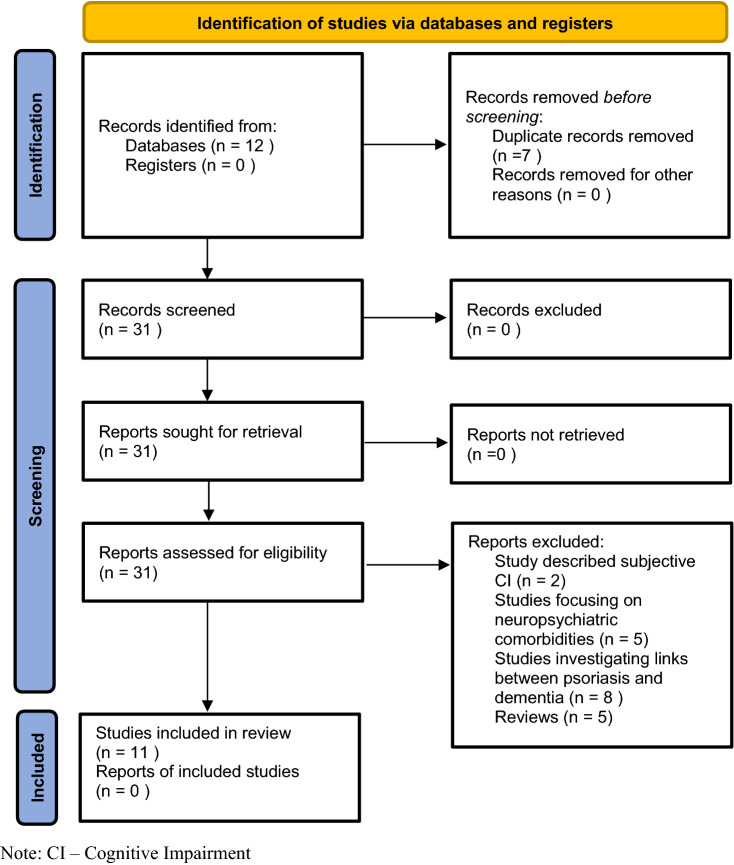
Flow diagram of studies identified, excluded and included in the systematic review. *CI* cognitive impairment

Based on the presence of the searched-for or synonymous terms, 38 articles were identified and subjected to further analysis. The full texts were completely analyzed to avoid the risk of missing potentially important data. The process for selecting articles in this phase is shown in Fig. 1.

The criteria for further inclusion in the analyses were: (1) the article must describe cognitive functioning in people with psoriasis; (2) the study must have been conducted on adults; and (3) it must have used objective neuropsychological methods. As a result of the research, articles meeting the criteria were identified, which were published between July, 2011 and March, 2021.

### Data extraction

DP and KWP extracted data from each study independently. The variables of interest for systematic review were: year of publication, country of origin, sample size, basic sociodemographic data (sex, age, education), clinical data (disease severity, disease duration), method of assessing cognitive functioning, and the main findings of the study. For meta-analyses, we collected data on the means and standard deviations (for both experimental and control groups) of the results of tests that were used in at least two different studies, criteria for the diagnosis of cognitive impairment adopted in a given study, and the frequency of cognitive impairment in the tested sample. During data extraction, it was found that there were major differences in how data were reported. For tools that were used in at least two studies, when the reports did not provide information on means and standard deviations, the authors were contacted via email and ResearchGate.net (where possible). It was impossible to perform a meta-regression as, due to the lack of responses to our inquiries, there were very large lacunae in variables that could be potential moderators and there were not many studies using each tool in which the results were properly described. Due to the low number of replications and the inability to control the moderators, the meta-analysis of the collected results was not carried out.

### Quality assessment

To evaluate the quality of the selected studies, we used an adapted version of the Newcastle–Ottawa cohort scale for case–control studies, which takes into consideration the selection of samples, comparability of subgroups and exposure. Using this scale, we scored each study independently. Inter-rater compatibility was satisfactory, and scoring differences were reconciled through discussion (Supplementary table 1).

### Ethics

As no contact was needed with participants to carry out this research, the consent of the Local Ethics Board was not required.

## Results

### Results of the systematic review

The search using the methodology described above yielded 11 studies that fulfilled all selection criteria (Table 1). In total, 971 patients with psoriasis participated in these studies (474 female and 497 male) and there were 10,242 controls. The mean age of the participants ranged from 40.3 [18] to 66.86 [19] years.

**Table 1:** Systematic review of included studies

Authors	Sample size/diagnosis of cognitive impairment	Number in control group/selection criteria	Basic sociodemographic characteristics	Clinical Characteristics	Names of the applied assessment tools	Definition of cognitive impairment	Main findings	Psychopathological symptoms: measure/main findings
Felipo et al. (2011). Spain [20]	21/0	54/ no information about selection criteria	Women constituted 61.9% of the group; mean age: 50 ± 15 years; no information about the education was provided	No information	PHES (DST, NCT-A & B, SD, LTT)	Adjusting for age and education level by means of normality tables, patients were classified as having mild cognitive impairment when the score was less than − 4 points	Inflammation or hyperammonemia alone does not induce cognitive impairment, but combination of their certain levels of is enough to induce cognitive impairment	Not analyzed
Marek et al. (2011). Poland. [21]	97	28 /no information about selection criteria	Women constituted 36.1% of the group; mean age: 44,1 ± 13,0 years; no information about patient's education	No information about patient's disease duration; mean disease activity assessed with PASI: 21.9	TMT A & B, Stroop test	No information	Patients achieve worse results than controls in working memory processes and executive functions	BDI-I; clinically significant depressive symptoms in 58 patients (> 12 points). No information about relationship between cognitive functioning and depressive symptoms
Gisondi et al., (2013). Italy [22]	41/18	37 / age, body mass index, level of education, smoking and alcohol consumption habits, prevalence of diabetes, hypertension, and hypercholesterolemia	Women constituted 36.6% of the group; mean age: 60 ± 5.3 years; mean years of education 8.6 ± 3.7	Mean disease duration in years: 15.4 ± 12; mean disease activity assessed with PASI: 15.5 ± 5.5	AVLT (immediate, delayed, Digit Span Forward & Backward, WST, FAB, ToL, AMT, TMT A & B, Raven’s 47 Progressive Colored Matrices, freehand copying and copying drawings with landmarks, Phonemic and Semantic Fluency Test	(1) subjective complaint of a memory (or other cognitive) deficit, confirmed by a relative or caregiver, (2) pathological performance on neuropsychological tests investigating a single or multiple cognitive domains, (3) normal performance of daily living activities measured with ad hoc scales and (4) no dementia	Cognitive impairment was observed in areas of long-term verbal memory, executive functions and attention	BDI; none of the cases had symptoms of depression according to the BDI
Colgecen et al. (2016). Turkey [18]	77	83 /age and sex	Women constituted 55.8% of the group; mean age: 40.3 ± 10.1 years; high school education or greater: 45.5%	Median disease duration in years: 10; median disease activity assessed with PASI: 15 (range: 12–17.7); scores < 10: 17 (22.1%) and ≥ 10: 60 (77.9%)	MoCA	No information	Cognitive impairment was observed in visuospatial domain and executive functioning. Presence of psoriasis, education level and area of residence were independently associated with cognitive impairment in patients and controls	BDI; All participants who scored > 17 were diagnosed as having depression and excluded from the study pool
Marek-Józefowicz et al. (2017). Poland [23]	97	91/ no information about selection criteria	Women constituted 53.6% of the group; mean age: 44.1 ± 13.0 years; no information about patient's education	No information about patient's disease duration; mean disease activity assessed with PASI: 21.9 ± 9.4	TMT A & B, Stroop test	No information	Compred to controls, patients present impairment of working memory	BDI-I; No information about relationship between CI and depressive symptoms
Innamorati et al. (2018). Italy [24]	50	50 / sex and age	Women constituted 44% of the group; mean age: 42.02 ± 12.16 years; high school education or greater: 34%	Mean disease duration in years: 16.36 ± 14.17; mean disease activity assessed with PASI: 4.56 ± 2.25	MMSE, AVLT (immediate, delayed), TMT A & B, Attentive matrices, Digit Span Forward & Backward, Clock test, Phonemic fluency test, Stroop test	No information	Patients (compared to controls) performed worse on most of the analyzed cognitive tests; cognitive impairment was independently associated with psoriasis even after controlling for psychopathology and alexithymia	HADS; patients with psoriasis reported more anxiety and depression than controls. No information about relationship between CI and depressive symptoms, but cognitive functioning components were not associated with physical and mental health components of Quality of Life
Pezzolo et al., (2018). Netherlands* [19]	*N* = 318*/ 25/219	9678 / age and sex	Women constituted 55.6% of the group; mean age: 66.86 ± 8.89 years; high school education or greater: 60.2%	76.7% of participants had mild psoriasis and moderate-to-severe psoriasis was diagnosed in 23.2% participants. No information about disease duration included in text	MMSE, AVLT (immediate, delayed, recognition), Stroop test, (reading, color naming, interference), LDST, Verbal fluency (animals), PPT	Presence of subjective cognitive complaints, objective cognitive impairment, and absence of dementia. Participants were classified as having MCI if they scored within 1.5 standard deviations of the age-adjusted and education-adjusted mean of the study population	Cognitive test scores and volumetric, microstructural, focal measures on brain MRI did not differ between psoriasis and nonpsoriasis participants. Results indicate that psoriasis was not associated with cognitive impairment	Not analyzed
Deveci et al. (2019). Turkey [25]	37	37 / sociodemographic characteristics	Women constituted 70.3% of the group; mean age: 42 ± 22 years; mean years of education 10 ± 5	Disease duration in years: ≤ 4 years (*n* = 5); 5–9 years (*n* = 8); ≥ 10 (*n* = 27); disease activity assessed with PASI 0–1 (*n* = 5); 1–3 (*n* = 17); 3–5 (*n* = 12); > 5 (*n* = 3)	Phonemic Verbal Fluency Test, AVLT (Öktem Verbal Memory Processes Test), ACTT, WCST	No information	Independently of depression, inflammation and oxidative stress levels, psoriasis patients have higher risk factors for cognitive impairment	BDI-I, BAI; no significant relationship between BDI, BAI scores and cognitive test scores
Di Carlo et al. (2020). Italy [26]	96/47	48 / age and education	Women constituted 39.6% of the group; mean age: 52.7 ± 11.7 years; mean years of education 13.06 ± 3.75	Mean disease duration in years: 9.61 ± 8.68 (PsA) 14.93 ± 13.67 (psoriasis); mean disease activity assessed with PASI: 0.90 ± 1.73	MoCA	Score < 26 indicates the presence of MCI	MCI is present in a significant proportion of PsA patients and is mainly determined by age, cutaneous variables, and disability	PsAID; no correlation between MoCA results and depressive symptoms
Padma et al. (2020). India [27]	100	100/ no information about selection criteria	Women constituted 36% of the group; age group of 31–40 years constituted the major part. 42% of subjects in the age group of 21–40 years; high school education or greater 26%	Disease duration ≤ 1 year: 18% 1–5 years: 44%; 5–10 years: 33%; > 10 years: 5%. No information about disease severity included in text	MMSE, BCRS	no information	Patients with psoriasis had cognitive deficits in the domains of attention, concentration, and total scores of SMMSE and BCRS	Not analyzed
Garcia et al. (2021). Brazil [28]	37 (PsA)/34	36 / age, educational attainment, and sex	Women constituted 46% of the group; mean age: 57.37 ± 13.48 years; high school education or greater: 5.4%	No information	MoCA	MoCA score < 26. To control for educational status, 1 point added to the MoCA score in patients with < 12 years of education	PsA patients may be at risk of dementia	HADS; no difference between the groups regarding the HADS general and subscales scores

*-234 psoriatic patients and 7137 reference subjects undergo neuropsychological testing and prevalence of MCI was demonstrated for 219 psoriasis patients. *MMSE* Mini-Mental State Examination, *AVLT* Auditory Verbal Learning Test, *LDST* Letter-Digit Substitution Task, *PPT* Purdue pegboard test, *MCI* Mild Cognitive Impairment, *MRI* Magnetic Resonance Imaging, *PASI* Psoriasis Area and Severity Index, *TMT* Trail Making Test, *WST* Weigl’s Sorting Test, *FAB* Frontal Assessment Battery, *ToL* Tower of London, *AMT* Attention Matrices Tests, *MoCA* *Montreal* Cognitive Assessment, *PHES* Psychometric Hepatic Encephalopathy Score, *DST* digit symbol test, *NCT-A* number connection test A, *SD* serial dotting test, *LTT* the line tracing test, *PsA* psoriasis arthritis, *HADS* Hospital Anxiety and Depression Scale, *BCRS* *Brief Cognitive* Rating Scale, *BDI* Beck Depression Inventory, *BAI* Beck Anxiety Inventory, *ACTT* Auditory Consonant Trigram Test, *WCST* Wisconsin Card Sorting Test, *PsAID* Psoriatic Arthritis Impact of Disease

The studies included in the review were cross-sectional, which limits the possible analyses and interpretations of the results as well as the factors responsible for the formation of CI and its dynamics. Level of education, as an important factor related to level of cognitive functioning, was taken into account when selecting the control group in only 3 studies, while in one study no data on possible differences between the groups were provided [25]. Many of the studies lacked basic information on the clinical course of the disease (e.g., [28]); while in the remaining studies, this information was presented in different ways ([19] vs [27]). The studies used different methodologies to assess CI level: MoCA was used in 3 studies; while, MMSE and AVLT were used in 2 studies (see Table 2 for a brief description of the tools).

**Table 2:** Brief description of tools used in more than 1 study

Assessment tool	Description	Cognitive processes assessed
Mini–Mental State Examination (MMSE)	Short screening tool	orientation to time, orientation to place, registration, attention and calculation, remote memory, nomination, repetition, stage command, writing, read and obey, and copy a diagram
Montreal Cognitive Assessment (MoCA)	Short screening tool	orientation, short-term memory/delayed recall, executive function/visuospatial ability, language abilities, abstraction, animal naming, and attention
Auditory Verbal Learning Test (AVLT)	AVLT a serial word learning task: 15 words are presented over five learning trials. After the fifth presentation, a sixth trial is administered with 15 new words. After the free recall of the second list, the patient is asked to recall the initial 15-word list. Retention is assessed with about 30 min delayed recall and recognition trials	verbal learning (immediate and delayed) free recall, recognition, retroactive and proactive interference

The analysis of the conducted studies also does not allow for an unambiguous determination of the frequency of CI in people with psoriasis: the criteria for diagnosis of CI in many of the studies analyzed were not clearly defined (e.g., [24]), and their levels ranged from 0 [20] to 91.9% [28].

The studies’ results indicated impaired cognitive functioning in people with psoriasis relative to people in the control group; however, these impairments pertained to many domains, preventing us from painting a clear description of the characteristics of these difficulties. The results showed irregularities in, among others, working memory processes [21, 23], executive functions [18, 21, 22], long-term verbal memory [22], attention [22, 27], and the visuospatial domain [28].

The large variety of research tools as well as the presentation of data about potential moderators make it very difficult to draw unambiguous conclusions about CI in this clinical group and prevents us from conducting a meta-analysis and meta-regression that would allow accurate quantitative assessment of CI severity and prevalence as well as the moderators thereof.

## Discussion

The article attempted to systematically review and meta-analyze studies on cognitive impairment in people with psoriasis. We identified 11 eligible studies. The obtained results suggest that cognitive impairment in people diagnosed with psoriasis is a major clinical problem, encompassing many cognitive domains. On one hand, these difficulties may have a negative impact on the quality of life of patients, further hindering their daily functioning. In addition, people with cognitive deficits may be more prone to misinterpretation of, for example, the reaction of their environment to the disease; however, this requires empirical research. Difficulties of this type may also adversely affect compliance with medical recommendations and the ability to continue work or study, but this hypothesis also requires empirical confirmation. It is an open question whether these difficulties are permanent and may turn into dementia in the future or whether are they limited in time and are due to, for example, cytostatic therapy.

First, attention should be paid to very large discrepancies between the obtained results. Studies show different orders of magnitude of differences in terms of cognitive impairment between people suffering from psoriasis and healthy people. Results differ greatly depending on the methods used (e.g., PHES [20], MMSE [19], or MoCA [28]) as well as the data characterizing the analyzed sample, such as level of education (high school education or greater in the Pezzolo sample [19] was 60.2% vs 5.4% in the Garcia et al. [28] sample). Unfortunately, we are not currently able to fully describe this phenomenon by, for example, determining sociodemographic factors (e.g., gender, age, education), clinical factors (severity of the disease, duration of the disease, treatment), or psychological factors (intensification of depressive symptoms, anxiety or stress), which could be moderators of such discrepancies, allowing for better estimates. In the first place, this inability is caused by the lack of replication of research and the use of different tools to assess the cognitive functioning of people with psoriasis. The second issue that needs to be addressed here is the very different and not always appropriate manners in which data were reported. In one of the studies [19], descriptive statistics were given for 318 patients (9678 controls), results obtained from cognitive tests were given for 234 (7173 controls), and MRI results for 62 patients (2664 controls). Unfortunately, here the data for the entire sample cannot be used as potential moderators due to the likely differences in scope between these 3 groups. In other studies either standard deviations were not reported [21, 23] or only the frequencies of diagnosed difficulties were given without mean values and standard deviations [18, 22]. Unfortunately, this information is not sufficient for a meta-analysis.

It is also worth emphasizing that there was a very large discrepancy in the frequency rate of cognitive impairment: these values ranged from 0 [19] to 91.9% [28]. For the reasons described above, it was also impossible to perform meta-analyses that would more accurately determine the frequency of cognitive impairments.

In the data extraction, apart from sociodemographic and clinical variables, the focus was also on the severity of depressive symptoms. However, in the analyzed studies, people with a high intensity of depressive symptoms (e.g., [18]) were either excluded from the study or it was pointed out that there was no relationship between the severity of depressive symptoms and cognitive impairment [25, 26]. Based on this information, it can be preliminary assumed that there is no relationship between depressive symptoms and cognitive decline in psoriatic patients, but if more studies are carried out on this topic, it should be verified by checking whether the severity of depressive symptoms is a moderator of heterogeneity in cognitive tests results. In future research, it is also worth considering a person-centered approach to the analysis of results, which could allow for a deeper analysis of the relationship between the above-mentioned factors.

### Limitations and further directions

This study has some limitations: there is not enough data to carry out meta-analyses on a sufficiently large amount of data or to identify potential moderators. Moreover, the analyses indicate issues that may be modified in further research: there should be greater consistency in methods used to diagnose cognitive impairment and, especially, in reporting data. For this purpose, recommendations prepared by the authors may prove helpful (see Appendix 1).

In addition to the above-mentioned limitations, an in-depth analysis of the texts indicated additional research directions that could be undertaken in the future. The collected data indicate the lack of longitudinal and prospective studies that could determine the role of individual factors in the formation and maintenance of cognitive impairment in psoriatic patients. In people diagnosed with psoriasis, it seems justified to conduct prospective studies on the influence of cytostatic treatment on cognitive functioning: in other groups of patients (e.g., those diagnosed with oncological diseases or rheumatoid arthritis), different types of treatment are used (e.g., surgical, hormonal) [29], which may additionally affect cognitive abilities. For psoriasis patients, this risk does not occur: in this case, it is also worthwhile to control variables related to severity of stress and depressive and anxiety symptoms. Due to the pharmacodynamics of methotrexate, such a study would require measurements at the start of treatment, 3 months later, and then again after 6 months to determine whether cognitive impairments remain at the same level.

Another direction for analysis could also be the use of a person-centered perspective (Latent Profile Analysis—cross-sectional data; trajectories—longitudinal studies), which would allow us to determine profiles of cognitive functioning and their possible determinants, or to distinguish subgroups characterized by different dynamics of change of CIs within a specific time period. Performing such analyses would allow for a deeper understanding of this issue, but at the same time would require a fairly large study group. In addition to controlling the objective indicators of CI (i.e., neuropsychological tests), it is also worth considering the determination of subjective difficulties in the field of cognitive functioning and their correlates, including psychological factors (such as cognitive appraisals and cognitive representations of the disease) and a broader analysis of the relationship between CI and indicators of adaptation to living with the disease, for example quality of life or satisfaction with life.

## Conclusions

The conducted analyses allow us to draw the following conclusions:

- Case–control studies on CI conducted with people with psoriasis use a variety of different measurement methods.
- There are no longitudinal studies that could help identify factors relevant to the development and maintenance of cognitive impairment in psoriasis. It would be worthwhile to conduct research that would help to better understand this phenomenon.
- Future research on this issue should have a unified methodology and data reporting method that allows for meta-analysis of the results and, thus, allow for a more comprehensive look at the problem of cognitive dysfunction in patients with psoriasis.

## Supplementary Information

Below is the link to the electronic supplementary material. Systematic Review was pre-registered at OSF—additional information will be made available after acceptance. https://doi.org/10.17605/OSF.IO/9ACJP

Supplementary file1 (DOCX 13 KB)

Appendix 1. Recommendations for reporting data from studies on cognitive impairment in chronic health conditions (DOCX 31 KB)
