# Supplementary material for: Cognitive impairment in psoriasis patients: a systematic review of case–control studies
Source: J Neurol. 2022 Aug 9;269(12):6269–78. doi: 10.1007/s00415-022-11317-2 (PMC9618480; doi:10.1007/s00415-022-11317-2)
Supplement: Supplementary file 1 — Supplementary file1 (DOCX 13 KB) [file 415_2022_11317_MOESM1_ESM.docx]

Supplementary table 1. Quality of the selected studies according to NOS criteria*

| Author (year) | Selection | Comparability | Exposure | Total scores |
| --- | --- | --- | --- | --- |
| Felipo et al. (2011) | 2 | 0 | 3 | 5 |
| Marek et al. (2011) | 4 | 0 | 3 | 7 |
| Gisondi et al., (2013) | 4 | 2 | 3 | 9 |
| Colgecen et al. (2016) | 2 | 2 | 3 | 7 |
| Marek-Józefowicz et al. (2017) | 4 | 0 | 3 | 7 |
| Innamorati et al. (2018) | 4 | 2 | 3 | 9 |
| Pezzolo et al., (2018) | 3 | 2 | 3 | 8 |
| Deveci et al. (2019) | 4 | 2 | 3 | 9 |
| Di Carlo et al. (2020) | 4 | 2 | 3 | 9 |
| Padma et al. (2020) | 2 | 0 | 3 | 5 |
| Garcia et al. (2021) | 3 | 2 | 3 | 8 |

* Wells GA, Shea B, O’Connell D, Peterson J, Welch V, Losos M, Tugwell P. (2000). The Newcastle-Ottawa Scale (NOS) for assessing the quality of nonrandomised studies in meta-analyses. 2000: http://www3.med.unipmn.it/dispense_ebm/2009-2010/Corso%20Perfezionamento%20EBM_Faggiano/NOS_oxford.pdf
