# Supplementary material for: Cognitive impairment in psoriasis patients: a systematic review of case–control studies
Source: J Neurol. 2022 Aug 9;269(12):6269–78. doi: 10.1007/s00415-022-11317-2 (PMC9618480; doi:10.1007/s00415-022-11317-2)
Supplement: Supplementary file 2 — Appendix 1. Recommendations for reporting data from studies on cognitive impairment in chronic health conditions (DOCX 31 KB) [file 415_2022_11317_MOESM2_ESM.docx]

**Recommendations for reporting data from studies on cognitive impairment in chronic health conditions.**

**Abstract**

Difficulties in cognitive functioning in people suffering from chronic diseases are an increasingly frequently analyzed phenomenon. Researchers are interested in, inter alia, the potential side effects of various types of treatment, the biological mechanisms of the emergence of such difficulties, and the potential roles of modifiable factors. However, the growth of this area should go hand in hand with the adoption of certain standards for reporting results and controlling potentially relevant variables. Thanks to the standardization of data reporting methods and the controlling of potential moderators, subsequent collective statistical analysis of such research may allow for a more accurate description of the phenomena in question. The following recommendations are intended to provide such standards and may also serve as a guide for people who are planning research in this area.

**Keywords**: Cognitive impairment; Guidelines; Chronic disease;

**Introduction**

In recent years, there has been an increasing number of studies on issues related to cognitive functioning in chronically ill people [1,2]. More and more studies analyze the relationship of cognitive impairment (CI) with both clinical factors, such as treatment [3], as well as psychological ones, such as intensification of depressive [4] or anxiety symptoms [5]. Due to the ever-increasing number of studies, it is important to develop recommendations to guide clinical work, thereby allowing us to determine the risk associated with the occurrence of CI or the selection of specific methods for diagnosing CI. Apart from the need to replicate the various methods used in CI studies, the potential moderators of this phenomenon remain an important issue – one that collective analysis could help shed light on.

Preparation of systematic reviews and meta-analyses on this subject has revealed a lot of issues related to reporting that are worthy of attention. For many studies, the manner in which the results are described do not allow them to be compared with other results. Consistency in the reporting of sample characteristics and research results will allow for a more detailed analysis of the factors responsible for the differences between results. Using, for example, meta-regression methods would allow us to investigate whether such differences may be due to differences in the structures of samples.

First of all, it is worth paying attention to a basic problem common to many manuscripts: in the descriptive statistics, information on the structure of the sample is given for the entire sample, while the CI studies were performed only on subgroups. In such cases, authors should either add additional columns to the sample description table or include such information in the Appendices. This seems particularly important because the percentage of responses from authors to queries is extremely low, and open access data sharing is not often used by authors for various reasons. Thus, some very interesting results cannot be included in summary analyses.

Another issue is the selection of basic sociodemographic and clinical variables. Regardless of the hypotheses and methods used in the study, it is worth collecting basic sample data about attributes that may be responsible for further potential differences in CI. Despite the fact that the list of such variables is very long and the ones mentioned in the guidelines are only very basic, further selection of variables is of course up to the researchers and data from previous studies.

In the case of CI, it is extremely important to give data on sex, age, and education. In the case of age, authors should report the mean age of the respondents (along with standard deviations). One common approach to reporting ages is to break the sample down into age ranges. When these ranges differ between papers, they are useless in further analyses: the widths of such ranges are different or the authors report only certain ranges, making them incommensurable. In the case of education, we recommend reporting both the frequency of people with a certain level of education and the average number of years of study. This will allow for a more in-depth analysis of the sources of possible heterogeneity of results related to education. The choice of other sociodemographic variables to be controlled in the research is left to the discretion of the researchers (in the literature, researchers have analyzed, inter alia, issues related to employment, ethnicity, and family status). A greater number of controlled variables will enable a more detailed analysis of the group; however, it may also be an additional burden for the respondents.

In the case of clinical variables in studies, it is worth collecting information on the duration of the disease (from the moment of diagnosis; means and standard deviations), the severity of the disease using a standardized tool, treatment (frequency), and comorbidity (frequency). For assessing severity, standardized, commonly-used scales should be chosen and the results should be described using M, SD, and frequency based on cut-off points (e.g., low intensity: XX%). An extremely important issue that is overlooked in many studies is comorbidity – in many cases, participants are diagnosed with more than one disease. Numerous studies show that CIs occur in many diseases (e.g., diabetes [6], COPD [7], rheumatoid arthritis [8,9], etc.), therefore studies should monitor whether patients have been diagnosed with other diseases. The final issue is the treatment used: we suggest reporting what proportion of a given group is undergoing a particular treatment. As in the above case, the clinical variables do not have to be limited to those mentioned above. It is also worth taking into account biological markers, research results using, e.g., evoked potentials, or assessing possible health behaviors, such as diet, physical activity, alcohol consumption, or smoking.

In terms of psychological variables, we would like to encourage authors to assess the severity of depression and anxiety symptoms in respondents. Numerous studies indicate a high prevalence of such symptoms in chronically ill people [10] and a relationship between these variables and CI. Researchers are encouraged to use, for example, short screening scales such as PHQ-9 (depressive symptoms)[11], GAD-7 (anxiety) [12], or HADS (both) [13], due to how quickly they can be administered and their ability to determine the frequency of clinically significant symptoms (using cut-off points). As in the case of sociodemographic and clinical variables, we also encourage researchers to analyze a wider range of variables in this group, such as fatigue.

Regarding the methods used to assess CI, we would like to encourage researchers to use screening tools in their research. This will allow the collection of more data with potential clinical applications. Screening methods have a great advantage in that they can be used by medical workers for quick screening, for example, during a doctor's visit. The use of many batteries or neuropsychological tests is limited to psychologists. In this case, the results of the MMSE [14] or MoCA [15] may prompt further, more in-depth neuropsychological diagnosis. We would also like to encourage researchers to report the incidence of CI, using cut-off points in the case of screening tools, and in the case of neuropsychological batteries, with precisely described diagnostic criteria. In studies that aim to compare different diagnoses with healthy controls or to assess relationships/predictors, we encourage authors to report both means and standard deviations of the results in each group.

The following guidelines may prove helpful not only in preparing articles, but also for planning research and selecting the most important variables and their operationalization (see table 1).

**PLEASE INSERT TABLE 1 HERE**

**Acknowledgments:** None

**Conflict of interests: None**

**References**

1. Kello N, Anderson E. Diamond B. Cognitive dysfunction in systemic lupus erythematosus: a case for initiating trials. Arthritis Rheumatol 2019;71(9):1413-1425. doi: 10.1002/art.40933

2. Kim HJ, Jung SO, Kim H, Abraham I, Systematic review of longitudinal studies on chemotherapy‐associated subjective cognitive impairment in cancer patients. Psycho-Oncol 2020;29(4):617-631. doi: 10.1002/pon.5339

3. Gibson EM, Nagaraja S, Ocampo A, Tam LT, Wood LS, Pallegar PN, ... Monje M. Methotrexate chemotherapy induces persistent tri-glial dysregulation that underlies chemotherapy-related cognitive impairment. Cell 2019;176(1-2):43-55. doi: 10.1016/j.cell.2018.10.049

4. Rubin LH, Maki PM, HIV, depression, and cognitive impairment in the era of effective antiretroviral therapy. Curr HIV/AIDS Rep 2019;16(1):82-95. doi: 10.1007/s11904-019-00421-0

5. Gulpers BJ, Voshaar RCO, van Boxtel MP, Verhey FR, Köhler S. Anxiety as a risk factor for cognitive decline: a 12-year follow-up cohort study. Am J Geriatr Psychiatry 2019;27(1):42-52. doi: 10.1016/j.jagp.2018.09.006

6. Biessels GJ, Despa F, Cognitive decline and dementia in diabetes mellitus: mechanisms and clinical implications. Nat Rev Endocrinol 2018;14(10):591-604. doi: 10.1038/s41574-018-0048-7

7. Yohannes AM, Chen W, Moga AM, Leroi I, Connolly MJ, Cognitive impairment in chronic obstructive pulmonary disease and chronic heart failure: a systematic review and meta-analysis of observational studies. J Am Med Dir Assoc, 2017;18(5):451-e1. doi: 10.1016/j.jamda.2017.01.014

8. Oláh C, Schwartz N, Denton C, Kardos Z, Putterman C, Szekanecz Z, Cognitive dysfunction in autoimmune rheumatic diseases. Arthritis Res Ther 2020;22(1):1-7. doi: 10.1186/s13075-020-02180-5

9. Pankowski D, Wytrychiewicz-Pankowska K, Janowski K, Pisula E, Cognitive Impairment in Patients with Rheumatoid Arthritis: A Systematic Review and Meta-analysis. Joint Bone Spine, 2021;105298. doi: 10.1016/j.jbspin.2021.105298

10. Birk JL, Kronish IM, Moise N, Falzon L, Yoon S, Davidson KW, Depression and multimorbidity: Considering temporal characteristics of the associations between depression and multiple chronic diseases. Health Psychol 2019;38(9):802-811. doi: 10.1037/hea0000737

11. Spitzer RL, Kroenke K, Williams JB, Patient Health Questionnaire Primary Care Study Group Validation and utility of a self-report version of PRIME-MD: the PHQ primary care study JAMA 1999;282:1737-1744. doi: 10.1001/jama.282.18.1737

12. Spitzer RL, Kroenke K, Williams JB, Löwe B. A brief measure for assessing generalized anxiety disorder: the GAD-7. Arch Intern Med 2006;166(10):1092-1097. doi: 10.1001/archinte.166.10.1092

13. Zigmond AS, Snaith RP. The Hospital Anxiety and Depression Scale Acta Psychiatr Scand 1983;67:361-370. doi: 10.1111/j.1600-0447.1983.tb09716.x

14. Folstein MF, Folstein SE, McHugh PR. MMSE: A practicalmethod for grading the cognitive state of patients for the clinician. J Psychiatr Res 1975;12:189-198.

15. Nasreddine ZS, Phillips NA, Bedirian V, et al. The Montreal Cognitive Assessment, MoCA: a brief screening tool for mild cognitive impairment. J Am Geriatr Soc 2005;53:695–699. doi: 10.1111/j.1532-5415.2005.53221.x

Table 1. Guidelines for reporting data in studies on cognitive impairment in chronic conditions: checklist

| Lp | Item | Yes | No* | *Reason |
| --- | --- | --- | --- | --- |
| 1 | Are descriptive statistics presented for the group in which CI was assessed? |  |  |  |
| Basic sociodemographic data | | | |  |
| 2 | Sex (N/%) |  |  |  |
| 3 | Age (M/SD) |  |  |  |
|  | Education |  |  |  |
| 4 | Years of schooling (M/SD) |  |  |  |
| 5 | N/% of participants with primary / secondary / etc. education |  |  |  |
| Basic clinical data | | | |  |
| 6 | Time since diagnosis (duration of the disease): M/SD |  |  |  |
| 7 | Severity of the disease (M/SD) |  |  |  |
| 8 | N/% of participants with low / moderate / etc. severity of the disease |  |  |  |
| 9 | N/% of participants using a specific type of treatment (including surgery or, e.g., radiotherapy) |  |  |  |
| 10 | N/% of people with a specific comorbid disease (e.g., hypertension - XX% etc.) |  |  |  |
| Basic psychological data | | | |  |
| 11 | Depressive symptoms (M/SD) |  |  |  |
| 12 | N/% of participants with low / moderate / etc, severity of the depressive symptoms. |  |  |  |
| 13 | Anxiety (M/SD) |  |  |  |
| 14 | N/% of participants with low / moderate / etc, severity of anxiety |  |  |  |
| Reporting data on cognitive functioning | | | |  |
| 15 | Was a screening tool used (e.g., MoCA/MMSE)? |  |  |  |
| 16 | Is the incidence of CI reported? |  |  |  |
| 17 | If the answer to the above question is yes, are the diagnostic criteria precisely reported (e.g., the number of domains that must be diagnosed as disturbed, the number of SDs below which the CI is diagnosed) |  |  |  |
| 18 | Is the M/SD reported for the test results assessing cognitive functioning in the analyzed group? |  |  |  |
| 19* | * in the case when the study scheme is planned to be compared with people with other diagnoses or healthy controls, are the Ms/SDs of the results of the tests assessing cognitive functioning reported? |  |  |  |
| 20* | * if the study design includes a comparison with people with other diagnoses or healthy controls, are descriptive statistics also provided for the compared groups in the main text or appendix (see points above)? |  |  |  |
